# Pneumatocele formation in a fatal adult pneumonia patient coinfected with *Streptococcus pyogenes emm*-type 3 and influenza A: a case report

**DOI:** 10.1186/s12879-020-05595-2

**Published:** 2020-11-26

**Authors:** Masahiro Sano, Aya Shimamoto, Nozomi Ueki, Motohiro Sekino, Hiroshi Nakaoka, Masahiro Takaki, Yoshiro Yamashita, Takeshi Tanaka, Konosuke Morimoto, Katsunori Yanagihara, Masahiro Nakashima, Kazuto Ashizawa, Koya Ariyoshi

**Affiliations:** 1 Department of Infectious Diseases, Nagasaki University Hospital, 1-7-1, Sakamoto, Nagasaki, 852-8501 Japan; 2 Department of Radiology, Nagasaki University Hospital, 1-7-1, Sakamoto, Nagasaki, 852-8501 Japan; 3 Department of Tumor and Diagnostic Pathology, Atomic Bomb Disease Institute, Nagasaki University Graduate School of Biomedical Science, 1-12-4, Sakamoto, Nagasaki, 852-8523 Japan; 4 Division of Intensive Care, Nagasaki University Hospital, 1-7-1, Sakamoto, Nagasaki, 852-8501 Japan; 5 Department of Clinical Medicine, Institute of Tropical Medicine (NEKKEN), Nagasaki University, 1-12-4, Sakamoto, Nagasaki, 852-8523 Japan; 6 Department of Laboratory Medicine, Nagasaki University Graduate School of Biomedical Sciences, 1-7-1, Sakamoto, Nagasaki, 852-8501 Japan; 7 Department of Clinical Oncology, Nagasaki University Graduate School of Biomedical Sciences, 1-7-1, Sakamoto, Nagasaki, 852-8501 Japan

**Keywords:** Pneumatocele, *Streptococcus pyogenes*, *emm*-type 3, Influenza a, Case report

## Abstract

**Background:**

A pneumatocele is a transient thin-walled lesion and rare complication in adult pneumonia.

A variety of infectious pathogens have been reported in children with pneumatoceles. We report the first case of adult pneumonia with pneumatocele formation that is likely caused by *Streptococcus pyogenes* and coinfection with influenza A virus.

**Case presentation:**

A 64-year-old Japanese man presented with a one-week history of fever, sore throat, and arthralgia. He was referred to our university hospital for respiratory distress. He required mechanical ventilation in the intensive care unit (ICU). Bacterial culture detected *S. pyogenes* in the bronchoscopic aspirates, which was not detected in blood. Although a rapid influenza antigen test was negative, an influenza A polymerase chain reaction (PCR) test was positive. Therefore, he was diagnosed with coinfection of influenza A and group A *streptococcus* (GAS) pneumonia complicated by probable streptococcal toxic shock syndrome. A chest radiograph on admission showed diffuse patchy opacification and consolidation in the bilateral lung fields.

Multiple thin-walled cysts appeared in both middle lung fields on computed tomography (CT). On the following day, the bilateral cysts had turned into a mass-like opacity. The patient died despite intensive care. An autopsy was performed. The pathology investigation revealed multiple hematomas formed by bleeding in pneumatoceles.

**Conclusions:**

There have been no previous reports of a pneumatocele complicated by *S. pyogenes* in an adult patient coinfected with influenza A. Further molecular investigation revealed that the *S. pyogenes* isolate had the sequence type of *emm3*.

## Background

A pneumatocele is a transient thin-walled lesion containing air in the interstitium of the lung secondary to the partial obstruction of the bronchial lumen and is usually seen in pediatric pneumonia. It is a rare complication in adult pneumonia, and often causes medical emergency such as tension pneumothorax [1–7]. A variety of infectious pathogens have been reported in children with pneumatoceles, such as *Staphylococcus aureus* [8], coagulase-negative *Staphylococcus* [8], *Streptococcus pneumoniae* [8], *Haemophilus influenzae* [8], *Klebsiella pneumoniae* [8], *Pseudomonas aeruginosa* [8], *Pneumocystis jirovecii* [9], *Enterobacter gergoviae* [10], *Mycobacterium tuberculosis* [11], *Enterobacter cloacae* [12], *Escherichia coli* [2], and hydrocarbon-induced pneumonia [8, 13]. The highly pathogenic *Streptococcus* species *S. pyogenes*, or group A *Streptococcus* (GAS), is not an uncommon cause of acute community-acquired pneumonia [14]. It is often characterized by the rapid onset of dyspnea and fever, with the predominant symptom of chest pain and is associated with high rates of pleural effusion and empyema [15]. A 6-year-old boy with a pneumatocele involving *S. pyogenes* was reported in 1961 [16], but to our knowledge, no adult case of pneumatocele involving *S. pyogenes* has previously been reported.

## Case report

### Clinical history

A 64-year-old Japanese man with a history of diabetes (HbA1c: 7.1%), hypertension, hyperlipidemia and gastric ulcer presented with a one-week history of fever up to 39.9 °C, sore throat, and arthralgia. He visited a local doctor and was prescribed an antipyretic after the confirmation of a negative rapid antigen test for influenza. Since his general condition continued to worsen for 4 days, he was referred to our university hospital for respiratory distress.

### Physical examination/laboratory data/images

**Fig. 1:**
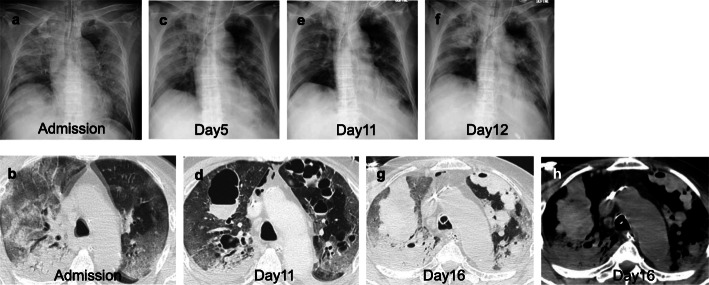
**a** Chest radiograph demonstrates diffuse ground glass opacities (GGOs) and consolidation in the bilateral lung fields. **b** CT shows widespread GGOs and consolidation in the dorsal lungs. **c**, **e** Follow-up chest radiographs reveal improvement in GGOs and consolidation in the upper and middle lung fields. Multiple thin-walled cysts appear in both middle lung fields (**e**). **d** On CT, GGOs and consolidation are improved, and multiple thin-walled cysts containing a small amount of fluid are seen in both upper lobes. **f** Bilateral cysts have turned into a mass-like opacity. **g**, **h** CT shows enlargement of the cysts and exacerbation of GGOs and consolidation in the background of the lungs. Fluid in the cysts show high attenuation, suggesting bleeding

His vital signs were a respiratory rate of 35/min, pulse rate of 130/min, blood pressure of 63/40 mmHg, body temperature of 37.2 °C, and oxygen saturation of 40% on room air. His state of consciousness with the Glasgow Coma Scale (GCS) was E3V4M5 (opened eyes in response to voice, confused/disoriented and localized to painful stimuli). Physical examination revealed an inflamed pharynx with swelling of the uvula and apparent coarse crackles in the right chest. Blood tests revealed leukopenia with a nuclear shift to the left, elevated C-reactive protein (CRP) and procalcitonin, severe respiratory failure (arterial blood gas pH 7.342, PaCO_2_ 26.0 mmHg, PaO_2_ 37.7 mmHg, HCO_3_^−^ 13.7 mmol/l), renal failure and metabolic acidosis (Table 1). A chest radiograph on admission demonstrated diffuse ground glass opacities (GGOs) and consolidation in the bilateral lung fields (Fig. 1a). Chest computed tomography (CT) showed widespread GGOs and consolidation in the dorsal lungs (Fig. 1b). He required mechanical ventilation (positive end-expiratory pressure: from day 1 to 2, 18 to 15 cmH_2_O; from day 3 to 8, 12 to 10 cmH_2_O; and from day 9, 8 cmH_2_O) in the intensive care unit (ICU).

**Table 1:** Laboratory Data on Admission(normal range)

**Haematology**	
Hb(11.4-14.8)	14.0g/dl
WBC(5000-8000)	2300/μl
Seg(40-60)	74.0%
Lymp(30.3-40.5)	21.0%
Mono(3.8-5.5)	1.0%
Eosino(0-4.5)	0.0%
Baso(0-1.9)	2.0%
At-Ly(0-2.0)	1.0%
Plt(18.0-35.0)	16.9×10^4^/dl
**Coagulation**	
PT-INR(0.84-1.14)	1.06
APTT(24.0-36.0)	38.2sec
AT-III(79-121)	105%
**Arterial blood gas (O**_**2**_ **face mask 12L/min)**	
pH(7.35-7.45)	7.342
PaCO_2_(35.0-45.0)	26.0mmHg
PaO_2_(80.0-100.0)	37.7mmHg
HCO_3_^-^(20.0-26.0)	13.7mmol/l
**Biochemistry**	
Na(135-148)	136mmol/l
K(3.50-5.30)	3.7mmol/l
Cl(98-106)	105mmol/l
BUN(8-22)	98mg/dl
Cre(0.4-0.7)	4.41mg/dl
TP(6.7-8.3)	6.8g/dl
Alb(4.0-5.0)	2.9g/dl
T.bil(0.3-1.2)	0.4mg/dl
AST(13-30)	126U/l
ALT(10-42)	41U/l
ALP(106-322)	433U/l
LDH(124-222)	1363U/l
CK(59-248)	671U/I
γGTP(13-64)	35U/l
Glucose(73-109)	374mg/dl
HbA1_c_(4.9-6.0)	7.1%
**Immunoserology**	
Rapid influenza antigen	negative
Procalcitonin(<0.06)	3.3ng/ml
CRP(0-0.14)	13.8mg/dl
**Culture**	
Sputum	*Streptococcs pyogenes* 1× 10^6^
Blood	negative

### Diagnosis

Bronchoscopic aspirates were positive for influenza A by polymerase chain reaction (PCR) at the time of admission and on the 4th day of hospitalization*.* Bacterial culture detected *S. pyogenes* in the aspirates, which was not detected in blood cultures repeated four times. He was diagnosed with coinfection of influenza A and GAS pneumonia complicated by probable streptococcal toxic shock syndrome (STSS). Because he had hypotension, renal dysfunction and acute respiratory distress syndrome (ARDS) without *S. pyogenes* bacteremia accompanied by concurrent or sequential infection with influenza A. We actually do not know which infection occurred first: influenza A or GAS pneumonia. Deoxyribonucleic acid (DNA) was extracted from the isolated *S. pyogenes* and tested by PCR targeting the *emm* region, which revealed the sequence type of *emm3*. Furthermore, the virulence genes *SpeA* and *SpeB* (but not *SpeC*) were detected.

### Hospital course

**Fig. 2:**
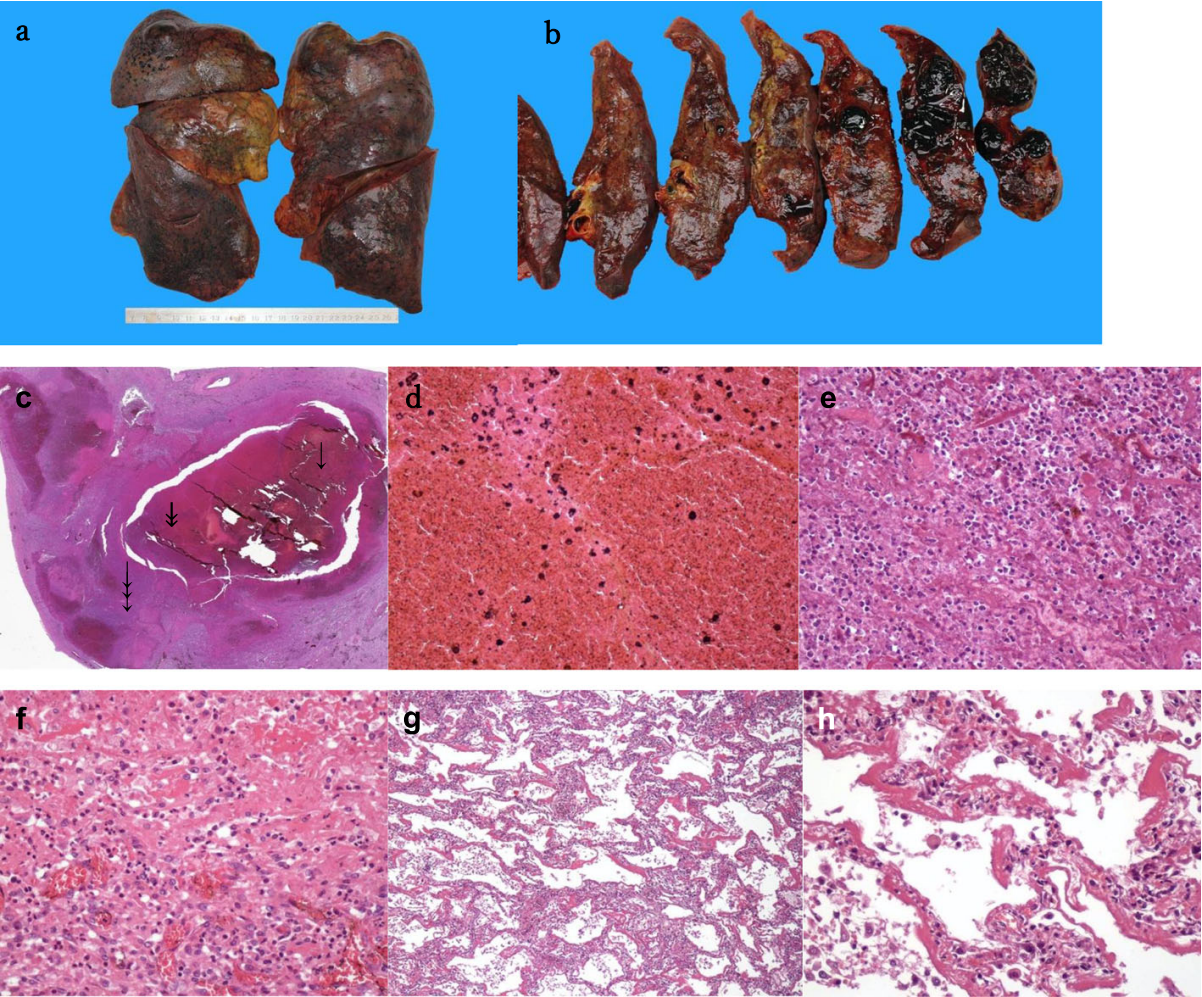
**a** Pathological findings. Macroscopically, multiple cysts with necrosis as heavy and hard lung. **b** Left lung hemorrhagic cystoid lesion in cut surface. **c** Hematoma, H.E. stain× 1. **d** Erythrocytes and fibrin, H.E. stain× 200 in arrow of Fig. 2c. **e** Necrotic tissue, H.E. stain× 100 in two head arrow of Fig. 2c. **f** Capillary vessel and fibroblast in the granulation tissue, H.E. stain× 200 in the three head arrow of Fig. 2c. **g**, **h** DAD H.E. × 200

Empirical intravenous antibiotic therapy was immediately initiated as follows: meropenem 1 g q12 h, linezolid 600 mg q12 h, levofloxacin 500 mg q48 h and peramivir 600 mg once. Hydrocortisone sodium succinate at 200 mg/day and pressor agents, including noradrenaline and pitressin, were administered to maintain hemodynamics. He was further supported with continuous hemodiafiltration (CHDF), extracorporeal membrane oxygenation (ECMO) and polymyxin B-immobilized fiber column direct hemoperfusion (PMX-DHP). On day 8, the administered antimicrobial agents were downgraded to ampicillin-sulbactam, clindamycin and levofloxacin following sensitivity results (Table 2). Because the patient’s condition was still fragile, we did not narrow the antibiotic spectrum. Follow-up chest radiographs (day 5; Fig. 1c) revealed improvement of GGOs and consolidation in the upper and middle lung fields. On day 11, multiple thin-walled cysts appeared in both middle lung fields on CT (Fig. 1e). The thin-walled cysts in both upper lobes contained a small amount of fluid (Fig. 1d). On the following day, the bilateral cysts had turned into a mass-like opacity (Fig. 1f) Chest radiograph on day 15 and CT on day 16 showed enlargement of the cysts and exacerbation of GGOs and consolidation in the background of the lungs (Fig. 1g). Fluid in the cysts showed high attenuation, suggesting bleeding (Fig. 1h). On day 22, multiple organ failure was considered irreversible, and the use of ECMO was terminated. The patient died on day 26. An autopsy was performed. The pathology investigation revealed multiple hematomas formed by bleeding in pneumatoceles on the surface and infiltration of massive inflammatory cells and erythrocytes in the necrotic lung tissue. In addition, vascular destruction was observed. Diffuse alveolar damage (DAD) was widely observed in images of the lungs. The boundary between the hematoma and granulation tissue was unclear, and no covering was found (Fig. 2). No bacteria grew in the cultures of the lung lesions.

**Table 2:** Streptococcus pyogenes (1×10^6^) MIC(μg/ml)^*^ *Minimum inhibitory concentration

Daptomysin	≦0.25
PenicillinG	≦0.015
Ampicillin	≦0.5
Ampicillin-sulbactam	≦0.5
Cefotaxime	≦0.5
Ceftazidime	≦0.5
Ceftriaxone	≦0.5
Cefepime	≦0.5
Meropenem	≦0.06
Clarithromycin	≦0.5
Clindamycin	≦0.12
Vancomycin	0.5
Levofloxacin	≦0.5
Linezolid	≦1

## Discussion and conclusion

To our knowledge, this is the first case report of fatal adult pneumonia with pneumatocele formation in which coinfection consisting of *S. pyogenes* infection and influenza A is thought to have played a role in the pathology.

### *S. pyogenes* pneumonia and its genetic characteristics

Nelson et al. summarized the case fatality ratio (CFR) for 1509 patients with GAS pneumonia of all ages and revealed a CFR of 27.9% for elderly individuals aged 65 years and older, which was much higher than 4.4 and 13.8% for children under 10 years of age and younger adults, respectively [17]. Although there was no evidence of bacteremia, our patient was elderly, and we detected virulence genes, including the streptococcal pyrogenic exotoxin genes *SpeA* and *SpeB*, which stimulate T cells as superantigens, resulting in nonspecific T cell overactivation and massive cytokine release. This process causes tissue damage, organ failure, and septic shock. The isolates in our case were inferred to carry *emm3*. There seems to be a tendency for serotypes 1 and 3 to be associated with life-threatening infections [17]. Matthias et al. analyzed a large number (*n* = 719) of *S. pyogenes* infections, including meningitis, erysipelas, necrotizing fasciitis, endocarditis, osteomyelitis and pneumonia, and recognized *emm3* as significantly associated with complications of respiratory distress in patients [18]. Despite the negative results of the rapid influenza antigen test, the more sensitive influenza type A PCR was repeatedly positive. We considered this to have had a pathogenic effect. A recently published paper reviewed that underlying influenza A virus infection provokes invasion of GAS, which leads to a fatal condition more often than influenza A infection alone in their mouse model [19].

### Necrotizing pneumonia or pneumatoceles and the timing of its appearance

According to a previous report, pneumatoceles appear 5–6 days after hospitalization in both children and adults [20]. However, in our case, cystic lesions were not present on day 5 and were noted first on day 11. They were initially assumed to be cavities caused by the toxins produced by *S. pyogenes* destroying the lung tissue, but later CT scan findings indicated thin-walled smooth-edged lesions. Pneumatoceles are considered to form as a consequence of the drainage of necrotic lung parenchyma, coupled with check valve and bronchiolar obstruction, which are caused by edematous luminal narrowing with inflammation [21]. Autopsy investigation showed pneumatoceles containing fluid, with necrosis around the pneumatoceles, fibrotic thickening of the alveolar septum and hyaline membranes in the air space. These findings indicate that the pathology was in the proliferative phase of DAD. We believe that this complex pathology arose because this patient survived substantially longer under modern life-support technology, such as CHDF and ECMO. This case report has some caveats. First, we investigated only *SpeA*, *SpeB*, and *SpeC* and no other *Spe* genes*,* such as *Spe G*, *H*, *I*, *J*, *K*, *L*, and *M*.

Second, we need to consider the correlation between hematoma and CHDF, which affects coagulation dysfunction.

## Data Availability

All data generated or analyzed during this study are included in this published article.

## Abbreviations

ICU: Intensive care unit
CT: Computed tomography
CRP: C-reactive protein
PCR: Polymerase chain reaction
GAS: Group A streptococcus
DNA: Deoxyribonucleic acid
GCS: Glasgow Coma Scale
EVM: Eye response, Verbal response, Motor response
GGO: Ground glass opacity
CHDF: Continuous hemodiafiltration
ECMO: Extracorporeal membrane oxygenation
PMX-DHP: Polymyxin B-immobilized fiber column direct hemoperfusion
STSS: Streptococcal toxic shock syndrome
ARDS: Acute respiratory distress syndrome
DAD: Diffuse alveolar damage
CFR: Case fatality ratio
